# Integrated metabolomics and transcriptomics analysis during seed germination of waxy corn under low temperature stress

**DOI:** 10.1186/s12870-023-04195-x

**Published:** 2023-04-10

**Authors:** Feng Jiang, Shishi Lv, Zili Zhang, Qingchun Chen, Jiaqi Mai, Xiaorong Wan, Pengfei Liu

**Affiliations:** 1 Zhongkai University of Agriculture and Engineering, Guangzhou, 510225 China; 2 Guangzhou Key Laboratory for Research and Development of Crop Germplasm Resources, Guangzhou, China

**Keywords:** Waxy corn, Low temperature stress, Seed germination, Transcriptome, Metabolome

## Abstract

**Background:**

Waxy corn has a short growth cycle and high multiple cropping index. However, after being planted in early spring, late autumn and winter, it is susceptible to low temperature (LT), which reduces the emergence rate and yield. Therefore, it is important to analyze the response mechanism of waxy corn under LT stress.

**Results:**

All phenotype indexes of waxy corn inbred lines N28 were significantly higher than waxy corn inbred lines N67 under LT. With the increase of LT stress time, all physiological indexes showed an upward trend in N28 and N67. Differentially expressed genes (DEGs) 16,017 and 14,435 were identified in N28 and N67 compared with nongerminated control under LT germination, respectively, and differential metabolites 127 and 93 were detected in N28 and N67, respectively. In addition, the expression level of some genes involved in plant hormones and mitogen activated protein kinase (MAPK) signaling pathways was significantly up-regulated in N28. Compared with N67, flavonoid metabolites were also significantly enriched in N28 under LT germination.

**Conclusion:**

Under LT stress, the inbred lines N28 was significantly higher than the inbred lines N67 in the phenotypic and physiological indices of cold resistance. Compared with N67, the expression levels of some genes involved in the plant hormones and MAPK pathways were significantly up-regulated in N28, and flavonoid metabolites were also significantly enriched in N28 under LT stress. These genes and metabolites may help N28 to improve cold resistance and may be as potential target genes for cold resistance breeding in waxy corn.

**Supplementary Information:**

The online version contains supplementary material available at 10.1186/s12870-023-04195-x.

## Background

Maize (*Zea mays L*.) can be divided into three types based on the starch composition of the endosperm in the seed, normal corn, waxy corn and sweet corn [1]. Waxy corn (*Zea mays L.sinensis Kulesh*), artificially bred after mutation of corn, is favored by many consumers [2]. It has high value because of its rich nutrition and unique flavor. Maize is one of the most important thermophilic crops and is vulnerable to low temperature (LT). Due to unstable temperatures in the early spring in China, crops are often subjected to sudden LT after sowing [3]. LT has a huge impact on crop growth, productivity and quality, etc. [4].

The process of seed germination is a series of biological changes from the absorption of water to the extension of hypocotyl. Corn seeds are affected by a series of factors during the germination process, and temperature is one of the important external conditions that affect seed germination. LT is one of the main abiotic stresses affecting the growth, development, and spatial distribution of plants [5] and tends to reduce and delay the germination rate of seeds and even causes germination failure [6]. In order to resist LT stress, plants have developed many methods to balance stress-induced damage effects, such as increasing the content of proline, increasing the activity of detoxification substances or enzymes [7]. After being induced by LT, plants can activate the defense mechanism through the synthesis of antioxidants, the increase of intracellular osmotic protection substances, and the adaptation of physical structure to restore the balance of metabolism and substances in the plant [8].

Plant endogenous hormones play an essential role in seed germination [9]. Gibberellin (GA) is an important regulatory hormone that can break seed dormancy and promote seed germination [10]. Reactive oxygen species (ROS) can induce the expression of genes related to GA synthesis, thereby promoting seed germination [11]. The accumulation of abscisic acid (ABA) will have a significant inhibitory effect on seed germination. Under LT stress, ABA accumulated in seeds can inhibit the generation of ROS and the accumulation of ascorbic acid (ASC), thereby inhibiting the germination of rice seeds [12]. There is an antagonistic effect between ABA and GA, which can jointly regulate the germination and dormancy of seeds. At the same time, GA can reduce the content of ABA and promote the germination of seeds [13]. ABA can inhibit the seed germination and hinder the promotion of GA [14].

Cell membrane is the main site of freezing damage and cold acclimation. Membrane fluidity is very important for maintaining the functional activity of membrane proteins and membrane itself, and is directly affected by temperature [15]. Studies have shown that the damage of cell membranes is mainly caused by the unsaturation of fatty acids and lipid peroxidation in the cell under LT [16]. The higher the content of malondialdehyde (MDA) is caused by lipid peroxidation, the worse the cold resistance of plants will be [17]. Therefore, when plants are subjected to LT stress, fatty acid dehydrogenase can regulate fatty acid unsaturation to increase membrane fluidity that improve the cold resistance of plants [18]. At the same time, lipids are an vital part of cell membranes, which provide energy for plant seed germination and seedling growth [19]. Soluble sugars, as a combination of membrane lipids, have a certain effect on maintaining the stability of cell membranes [20].

There are a large number of genes in seeds that can be induced by LT and can quickly generate stress proteins to resist LT damage. For example, cytochromes P450 (P450s) are involved in the biosynthesis of brassinolide, and brassinolide can increase the cold tolerance and stress resistance of seedlings [21]. The BURP family genes have a positive effect on plants adapting to unfavorable and variable environments [22], for example, the BURP domain protein (*OsBURP16*) in rice can reduce pectin content and cell adhesion, while increasing non-sensitivity to biological stress [23, 24]. Transcription factors can activate the expression of target genes by recognizing partial promoter functional elements, causing changes in LT-related metabolic pathways [25]. Studies have shown that transcription factors include *AP2/ERF, NAC, WRKY, MYB, bZIP* and *ZFPs, etc.*. [26], which play a role in specifically binding to the cis-acting elements of target gene, thereby regulating the expression of downstream genes in response to LT signals. Moreover, downregulation of LT-related genes such as dehydration-responsive protein, heat shock protein 70, ethylene-responsive transcription factor, cold-regulated 413 plasma membrane protein, and LT-induced protein in plants is essential to adapting to LT [6]. Protein phosphatase 2Cs (PP2Cs) are a subfamily of protein phosphatases, which are widely involved in the transmission of stress signals. Studies have shown that overexpression of *ZmPP2C2* gene in maize can increase the LT tolerance of transgenic plants by activating the antioxidant system and reduce the accumulation of H_2_O_2_ in the cell [27], and the transcription level of *AtPP2Cs* is induced by LT stress [28].The response of plants to LT stress is the common result of expression of multiple genes, involving the expression of multiple genes, and having a complex regulatory network. Understanding the role and function of genes related to LT response will help improve the cold tolerance of plants. In the past, people have realized that there are genotypic differences in the cold tolerance of maize, and a lot of research has been carried out on its cold tolerance mechanism [29–33]. However, there are few studies on the genetic mechanism of LT tolerance during seed germination.

In this study, waxy corn inbred lines N28 and N67 were used as materials to measure the phenotype and physiological and biochemical substances of waxy corn during seed germination under LT treatment. Meanwhile, key candidate genes were found using the transcriptome sequencing and metabonomics technology, laying a theoretical foundation for breeding new varieties with LT tolerance.

## Results

### Phenotype variations during N28 and N67 germination

**Fig. 1:**
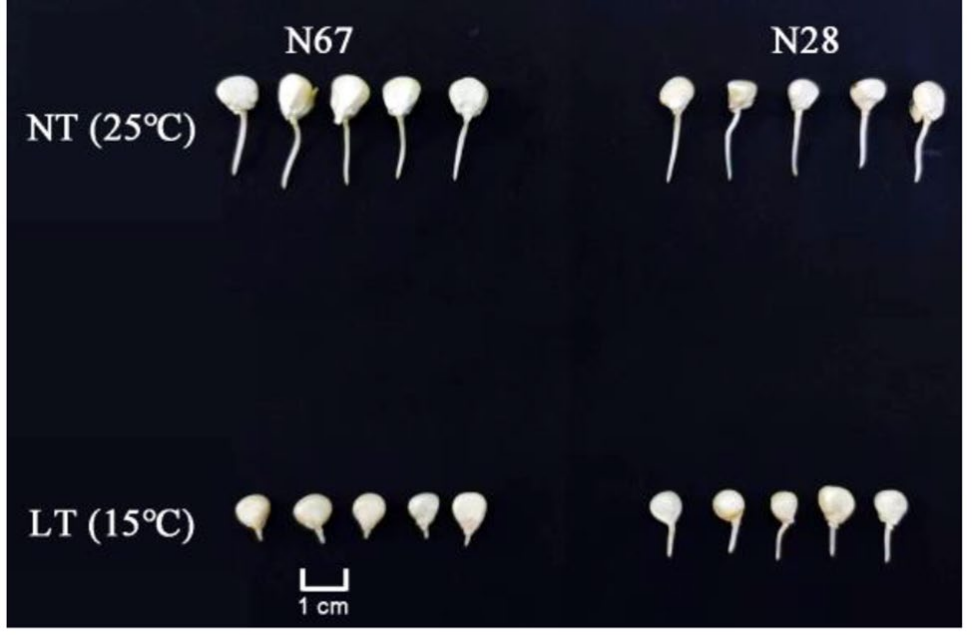
Phenotype of germinated seeds of N28 and N67 at NT (25 ℃) and LT (15 ℃) after 3 days of germination

The experimental materials N28 and N67 were germinated under the NT of 25 °C and the LT of 15 °C, respectively. After 3 days of germination, there was no significant difference in bud length between N28 and N67 at NT, but the difference was significant at LT (Fig. 1). Moreover, seed germination rate and energy of two inbred lines were normal under NT conditions, while under LT conditions, seed germination rate and energy dropped significantly. The results showed that cold resistance had a significant difference between N28 and N67 (Additional file 1: Table S1).

In order to analyze the phenotypic differences between N28 and N67 under LT germination, we detected some indexes of N28 and N67 including germination rate (GR), germination energy (GE), germination index (GI), vitality index (VI), root length (RL), bud length (BL), fresh weight (FW) and dry weight (DW) under NT and LT conditions. These indexes were analyzed by variance analysis (Table 1). As shown in the Table 1, there was no significant difference in the phenotype indexes of N28 and N67 under NT conditions, while there was a significant difference under LT conditions. Indexes of N28 were significantly higher than those of N67 under LT, such as GR, VI, BL, FW and DW, and GE, GI and RL. The above results showed that LT inhibited seed germination and cold tolerance of N28 was significantly greater than that of N67.

**Table 1:** Phenotypic variance analysis between N28 and N67

Processing group	Source of variation	GR (%)	GE (%)	GI	VI	RL (cm)	BL (cm)	FW (g)	DW (10^− 3^ g)
**NT**	Within Groups Variable	0.00	0.00	7.72	1742.04	65.69	2.63	7.31	14233.33
	Between Group Variable	0.00	0.00	0.06	107.36	0.81	0.20	1.03	19266.67
	* F-*value	3.66	0.22	0.01	0.68	0.37	5.13	1.42	4.46
**LT**	Within Groups Variable	0.05	0.06	1.88	1.04	0.36	0.01	0.04	34.41
	Between Group Variable	0.20	0.33	7.35	12.27	3.74	0.50	5.04	3611.31
	* F-*value	30.25^*^	196.00^**^	174.68^**^	23.09^*^	214.59^**^	33.53^*^	45.33^*^	94.61^*^

** indicates a extremely significant difference, * indicates a significant difference

### Effects of temperature on physiological index between N28 and N67 germination

**Fig. 2:**
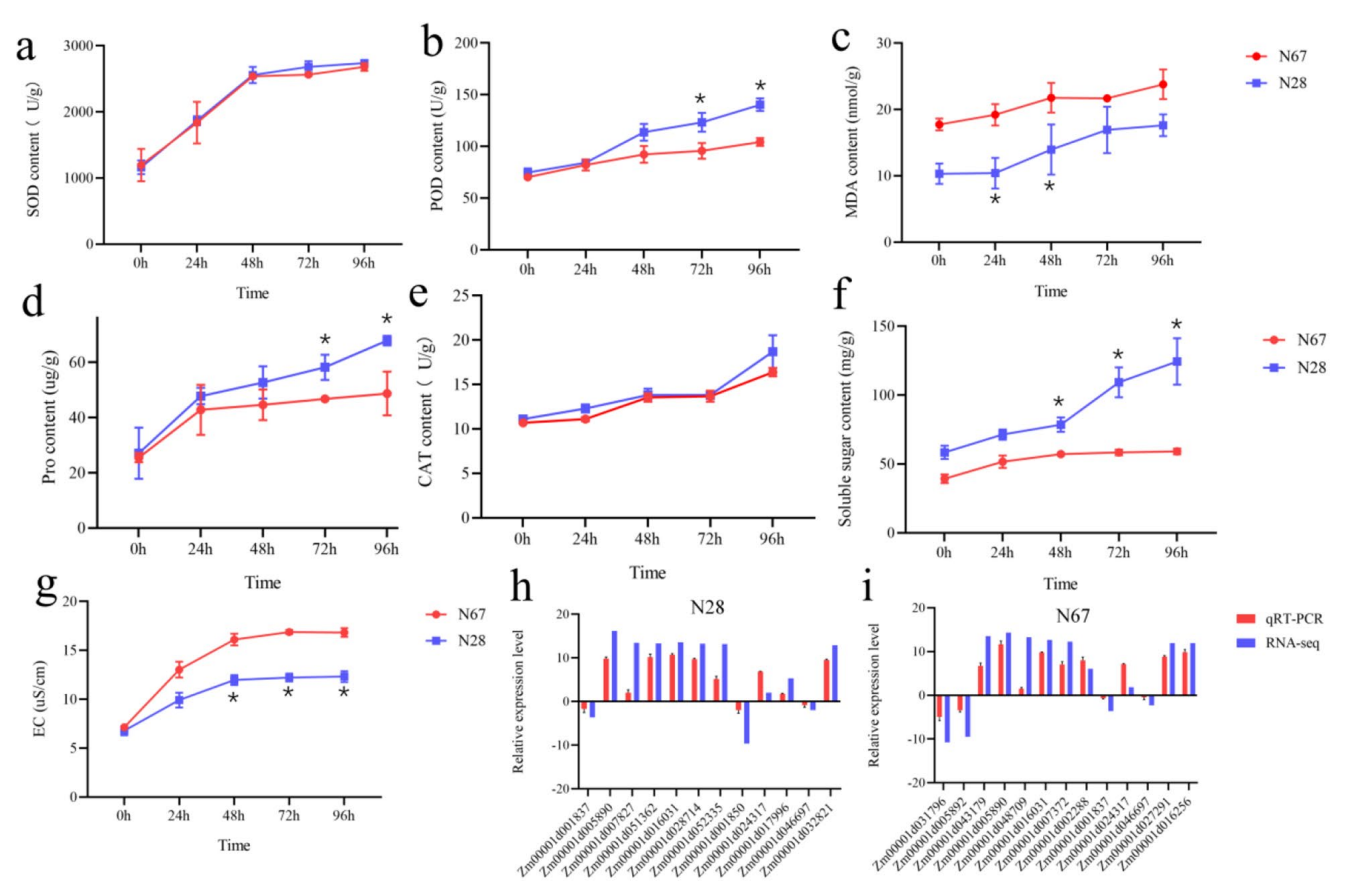
Physiological index and qRT-PCR analysis of N28 and N67. X-axis of **a**-**g** represents LT stress time. Y-axis of **a**-**g** represents substance content. X-axis of **h** and **i** represents gene name, Y-axis of **h** and **i** represents DEGs fold change value of MC28_VS_ML28 and MC67_VS_ML67, respectively. Three individual replicates were used to reduce the experimental error. The bars represent mean ± SE (n = 3). * means p-value < 0.05 between N28 and N67

Plant cells will undergo a series of changes to produce various substances used to resist or adapt to adversity, thereby maintaining normal physiological functions under adversity stress. N28 and N67 were germinated at 15 and 25 °C, respectively, and samples were taken after 0, 24, 48, 72 and 96 h of germination. We measured the changes in electronic conductivity (EC), malonaldehyde (MDA), superoxide dismutase (SOD), catalase (CAT), peroxidase (POD), proline (PRO) and soluble sugar (SS) content of N28 and N67 at different time under LT stress. All physiological indexes presented an upward trend with the extension of germination time (Fig. 2a-g). There was no significant difference in SOD and CAT under LT stress between N28 and N67; the content of soluble sugar (SS) and EC was significantly different after 48 h under LT stress; the content of POD and PRO was significantly different after 72 h under LT stress; the content of MDA was significantly different after 24 and 48 h under LT stress. It was found that N28 accumulated more protective substances than N67 under LT stress, and this might be the reason why N28 was more resistant to cold than N67.

### Transcriptome analysis for N28 and N67

In this study, 148.09 Gb Clean Data was obtained by transcriptome sequencing analysis of 18 samples, and the Clean Data of each sample was greater than 6Gb. In order to ensure the quality of analysis, the raw reads were filtered to obtain high-quality clean reads. We got a total of 9.9 × 10^8^ high-quality clean reads (Additional file 2: Table S2). High-quality clean reads were mapped to the maize B73 reference genome through HISAT2 software. In the present study, a total of 37,870 genes were identified in all samples, and 10,423 novel genes were detected. These newly identified genes still need further research to explore their functions in waxy corn. In order to truly reflect the expression level of the transcript, the number of mapped reads and the length of transcript in each sample need to be normalized. FPKM (fragments per kilobase of transcript per million mapped reads) was used as an index to measure the expression level of transcripts or gene.

### Analysis of differentially expressed genes (DEGs) and qRT-PCR

Gene expression is affected by the environment, and changes over time. We analyzed the DEGs of N28 and N67 under LT germination. Results are shown in Table 2. Compared with each nongerminated control, 9265 and 7948 DEGs were up-regulated, and 6752 and 6487 DEGs were down-regulated in N28 and N67 under LT germination, respectively. There were 5918 DEGs between N28 and N67 under LT germination, including 3261 up-regulated and 2657 down-regulated DEGs. These results also indicated that gene expression between N28 and N67 had significent difference under LT stress.

**Table 2:** The number of differential genes between comparison groups

Group	Total	Down	Up
MC28_vs_ML28	16,017	6752	9265
MC67_vs_ML67	14,435	6487	7948
ML67_vs_ML28	5918	2657	3261

MC28 and MC67 refer to nongerminated control samples of N28 and N67; ML28 and ML67 refer to N28 and N67 samples germinated at LT, respectively. Down represents down regulation. Up represents up regulation.

In order to verify the RNA-seq data, we selected 20 candidate DEGs for qRT-PCR. These DEGs are involved in plant hormone signal transduction, phenylpropanoid biosynthesis, flavonoid biosynthesis, amino acid synthesis, hydrogen peroxide decomposition, and lipid metabolism. It also included some membrane proteins genes and transcription factors genes. These DEGs included up-regulated and down-regulated genes between LT-treated sample and nongerminated control. The expression profiles of all candidate genes analyzed by qRT-PCR were in agreement with those obtained by RNA-seq (Fig. 2h and i), indicating that the RNA-seq results were reliable.

### GO annotation and enrichment analyses

**Fig. 3:**
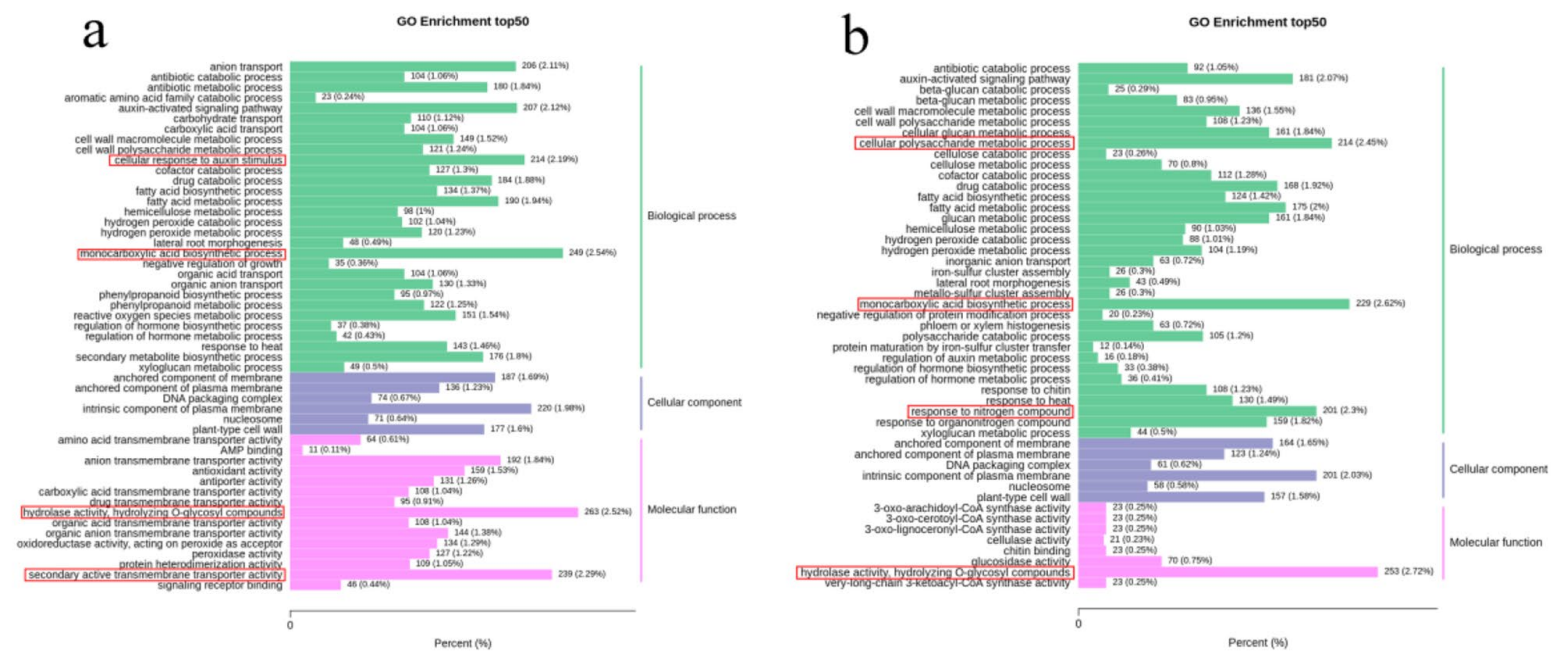
Go analysis of DEGs from N28 and N67. **a** Top 50 GO terms for MC28_VS_ML28. **b** Top 50 GO terms for MC67_VS_ML67. X-axis means percentage and number of DEGs. Y-axis represents GO terms. GO terms are mainly divided into three classes: cellular component, molecular function, and biological process

The Gene Ontology (GO) knowledgebase is the world’s largest source of information on the functions of genes. GO analysis is a foundation for computational analysis of large-scale molecular biology and genetics experiments. We identified 16,017 and 14,435 DEGs in N28 and N67 compared with their nongerminated control under LT germination. We analyzed these DEGs, to understand their regulation mechanisms. These DEGs are mainly divided into three classes of GO: cellular component, molecular function, and biological process. These DEGs are annotated into the GO database for functional annotation and enrichment analysis. The GO terms “monocarboxylic acid biosynthetic process”, “hydrolase activity, hydrolyzing O − glycosyl compounds”, “secondary active transmembrane transporter activity” and “cellular response to auxin stimulus” were the most highly enriched among gene sets in N28, and the GO terms “hydrolase activity, hydrolyzing O − glycosyl compounds”, “monocarboxylic acid biosynthetic process”, “cellular polysaccharide metabolic process” and “response to nitrogen compound” were the most highly enriched among gene sets in N67 (Fig. 3). N28 and N67 might have common regulation mechanisms under LT germination such as “monocarboxylic acid biosynthetic process” and “hydrolase activity, hydrolyzing O − glycosyl compounds”. The most DEGs were categorized in “monocarboxylic acid biosynthetic process” in N28, but the most DEGs were categorized in “hydrolase activity, hydrolyzing O − glycosyl compounds” in N67.

**Fig. 4:**
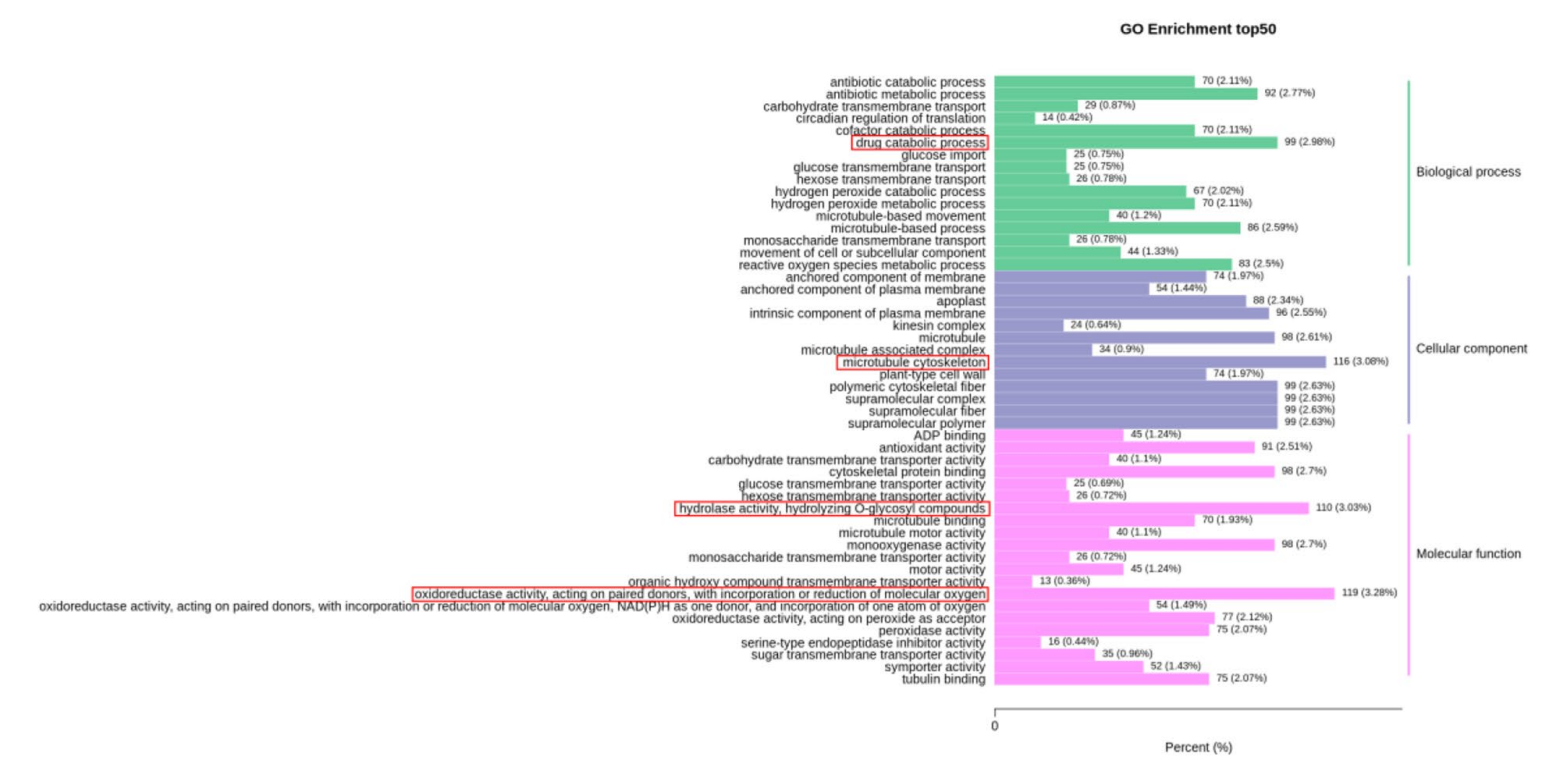
Go analysis of DEGs between N28 and N67 under LT stress. X-axis means percentage and number of DEGs. Y-axis represents GO terms. GO terms are mainly divided into three classes: cellular component, molecular function, and biological process

There were 5918 DEGs between N67 and N28 under LT germination. The GO terms “oxidoreductase activity, acting on paired donors, with incorporation or reduction of molecular oxygen”, “microtubule cytoskeleton”, “hydrolase activity, hydrolyzing O − glycosyl compounds” and “drug catabolic process” were the most highly enriched (Fig. 4). The highly enriched GO terms between N67 and N28 may cause the difference in resistance, but further research is still needed to clarify the mechanism of the difference in resistance.

### KEGG enrichment analysis of DEGs

**Fig. 5:**
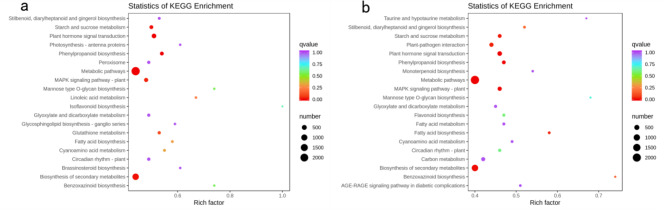
KEGG annotations and enrichment of DEGs from N28 and N67. **a** KEGG pathways for MC28_VS_ML28. **b** KEGG pathways for MC67_VS_ML67. Y-axis represents the KEGG pathway. X-axis represents Rich factor. The greater the Rich factor is, the greater the degree of enrichment is. The larger the point is, the greater the number of differential genes enriched in the pathway is. The redder the color of the dot is, the more significant the enrichment is

KEGG organically combines genomic information and high-level functional information to provide systematic analysis of big data generated by genome sequencing and other high-throughput sequencing. Compared with each nongerminated control, DEGs identified in N28 and N67 were analyzed by KEGG under LT germination. As shown in Fig. 5, “metabolic pathways”, “biosynthesis of secondary metabolites”, “plant hormone signal transduction” and “phenylpropanoid biosynthesis” were the most highly enriched different genes in N28, and “metabolic pathways”, “biosynthesis of secondary metabolites”, “plant hormone signal transduction” and “plant − pathogen interaction” were the most highly enriched different genes in N67. The same pathways such as “biosynthesis of secondary metabolites” and “plant hormone signal transduction” might greatly contribute to resisting adversity in N28 and N67. “MAPK signaling pathway − plant” and “starch and sucrose metabolism” were also enriched in N28 and N67. It was reported that different protein kinase families (such as MAPKs) are activated by osmotic stresses [34].These protein kinases and sucrose metabolism may play a vital role in the response of waxy corn to LT stress.

**Fig. 6:**
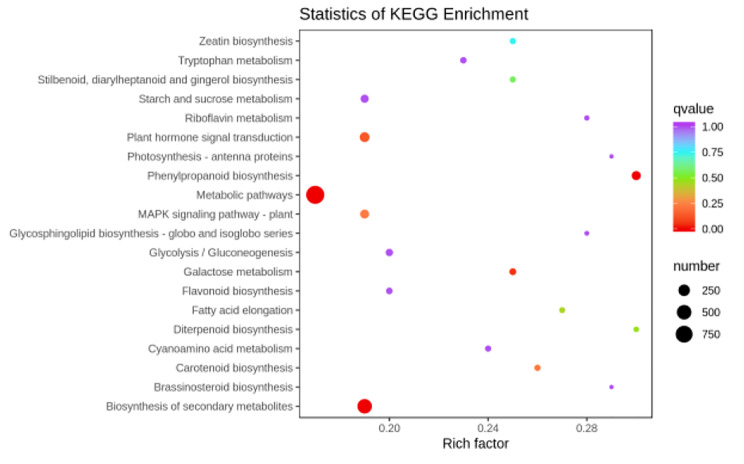
KEGG annotations and enrichment of DEGs between N28 and N67 under LT stress. Y-axis represents the KEGG pathway. X-axis represents Rich factor. The greater the Rich factor is, the greater the degree of enrichment is. The larger the point is, the greater the number of differential genes enriched in the pathway is. The redder the color of the dot is, the more significant the enrichment is

“Metabolic pathways”, “biosynthesis of secondary metabolites”, “phenylpropanoid biosynthesis” and “galactose metabolism” were the most significently enriched in ML67_VS_ML28 (Fig. 6). “Galactose metabolism” was not highly enriched in N67 and N28, but was more enriched in N28 than in N67 (Fig. 6). These pathways may be one of the reasons why the resistance of N28 to LT is higher than that of N67.

### Analysis of transcription factors (TFs)

**Fig. 7:**
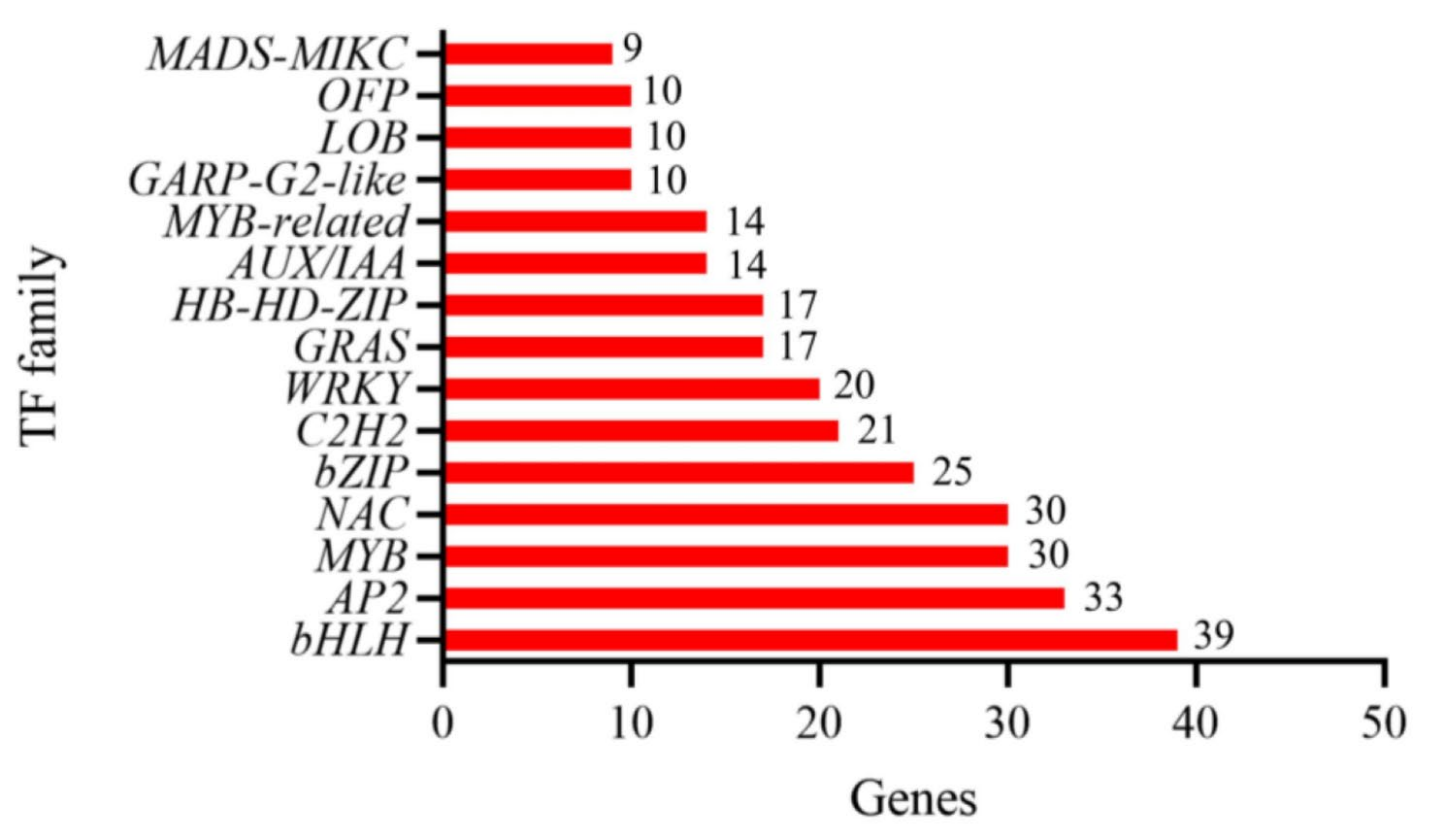
Top 15 TF families for DEGs from ML67_VS_ML28. X-axis represents gene number and Y-axis represents the top 15 transcription factor families

When plants face a harsh living environment, they will adjust the body’s physiological metabolic activity in a variety of ways, and the regulation of TFs is an important regulation method. Molecular and genetic studies have found many TFs that are important in regulating gene expression to enhance plant resistance when plants are stressed by adversity. In the present study, 447 differently expressed TFs were detected between N28 and N67 under LT germination, they were classified as 65 families and the top 15 of TF families are shown in Fig. 7. Among them, *bHLH, AP2, MYB, NAC* and *bZIP* account for 35.12% of the total number of TFs.

### Metabolome composition analyses

This research is based on the Ultra Performance Liquid Chromatography Tandem Mass Spectrometry (UPLC-MS/MS) detection platform and Maiwei’s self-built database. Compared with nongerminated control, 65 and 61 differential metabolites were up-regulated, and 62 and 32 differential metabolites were down-regulated in N28 and N67 under LT germination, respectively. 87 differential metabolites were detected between N28 and N67. Among these differentially accumulated metabolites, 45 were up-regulated, and 42 were down-regulated (Table 3).

**Table 3:** The number of differential metabolites between comparison groups

Group name	Total	Down	Up
**MC28_vs_ML28**	127	62	65
**MC67_vs_ML67**	93	32	61
**ML67_vs_ML28**	87	42	45

MC28 and MC67 refer to nongerminated control samples of N28 and N67; ML28 and ML67 refer to N28 and N67 samples germinated at LT, respectively; Down represents down regulation; Up represents up regulation.

**Fig. 8:**
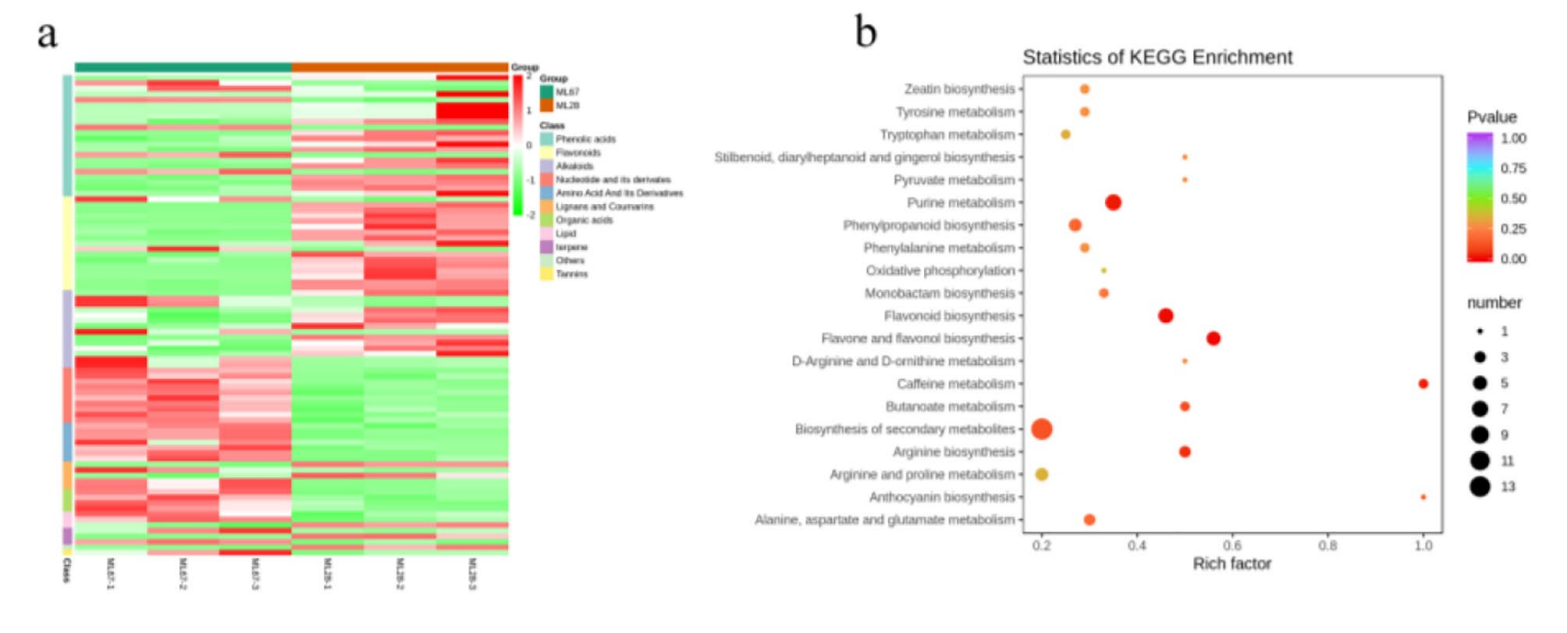
Clustering and KEGG analysis of differential metabolites between N28 and N67 under LT treatment. **a** Cluster heating maps of N28 and N67. The abscissa represents the sample name, and the ordinate represents the difference metabolites and hierarchical clustering results. ML28 and ML67 refer to N28 and N67 samples germinated at LT, respectively. 1 ,2 and 3 refer to the three replicates. Red means high content, green means low content. **b** KEGG analysis of N28 and N67. X-axis represents Rich factor. The greater the Rich factor is, the greater the degree of enrichment is. The larger the point is, the greater the number of differential genes enriched in the pathway is. The redder the color of the dot is, the more significant the enrichment is

In order to understand the resistance differences of N28 and N67 at the metabolic level under LT stress, the 87 differentially expressed metabolites produced by LT stress were clustered. The results showed that the relative content of phenolic acids and flavonoids in N28 was significantly higher than that of N67 in N28, but the relative content of nucleotide and its derivates, lignans and coumarins, amino acid and its derivatives and organic acids was significantly low in N28 (Fig. 8a). Therefore, content changes of above-mentioned metabolites might be one of the reasons why N28 was more resistant to cold. In summary, these differentially expressed metabolites play an important role in the response to LT stress.

Differential metabolites produced between N28 and N67 were also used for KEGG analysis under LT germination. As shown in Fig. 8b, “flavone and flavonol biosynthesis”, “flavonoid biosynthesis”, “purine metabolism”, “caffeine metabolism” and “arginine biosynthesis” were enriched differentially expressed metabolites between N67and N28; “flavone and flavonol biosynthesis” were the most highly enriched. These metabolic pathways may have a certain correlation with LT stress, but further research is needed to provide a research basis for the response mechanism of waxy corn under LT stress.

**Fig. 9:**
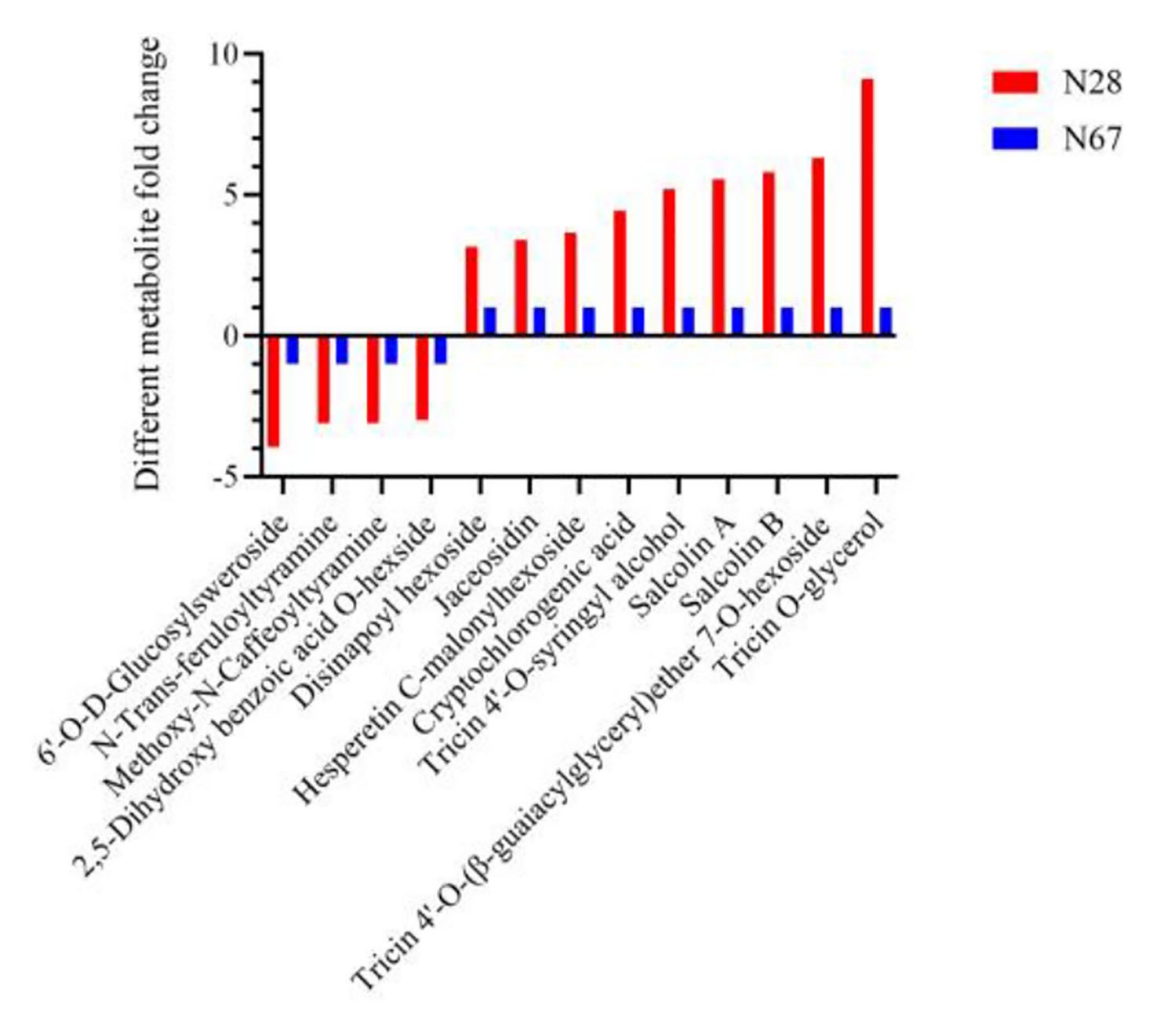
Analysis of key metabolites between N28 and N67 under LT treatment. X-axis represents types of differential metabolites. Y-axis represents differential metabolites fold change value

We further analyzed the key metabolites between N28 and N67. The log_2_^(fold_change)^ value of these key metabolites was greater than 3. As shown in Fig. 9, compared with N67, flavonoids and phenolic acid metabolites were significantly up-regulated, while terpenoid and alkaloid metabolites were significantly down-regulated in N28. Some metabolites were up-regulated more than 3 times, such as disinapoyl hexoside, jaceosidin, hesperetin C-malonylhexoside, cryptochlorogenic acid, tricin 4’-O-syringyl alcohol, salcolin A, salcolin B, tricin 4’-O-(β-guaiacylglyceryl)ether 7-O-hexoside and tricin O-glycerol. Some metabolites were down-regulated more than 3 times, such as 6’-O-D-glucosylsweroside, N-trans-feruloyltyramine, methoxy-N-caffeoyltyramine and 2,5-dihydroxy benzoic acid O-hexsid. Furthermore, tricin 4’-O-(β-guaiacylglyceryl)ether 7-O-hexoside and tricin O-glycerol were up-regulated 6-fold and 9-fold in N28, respectively.

**Fig. 10:**
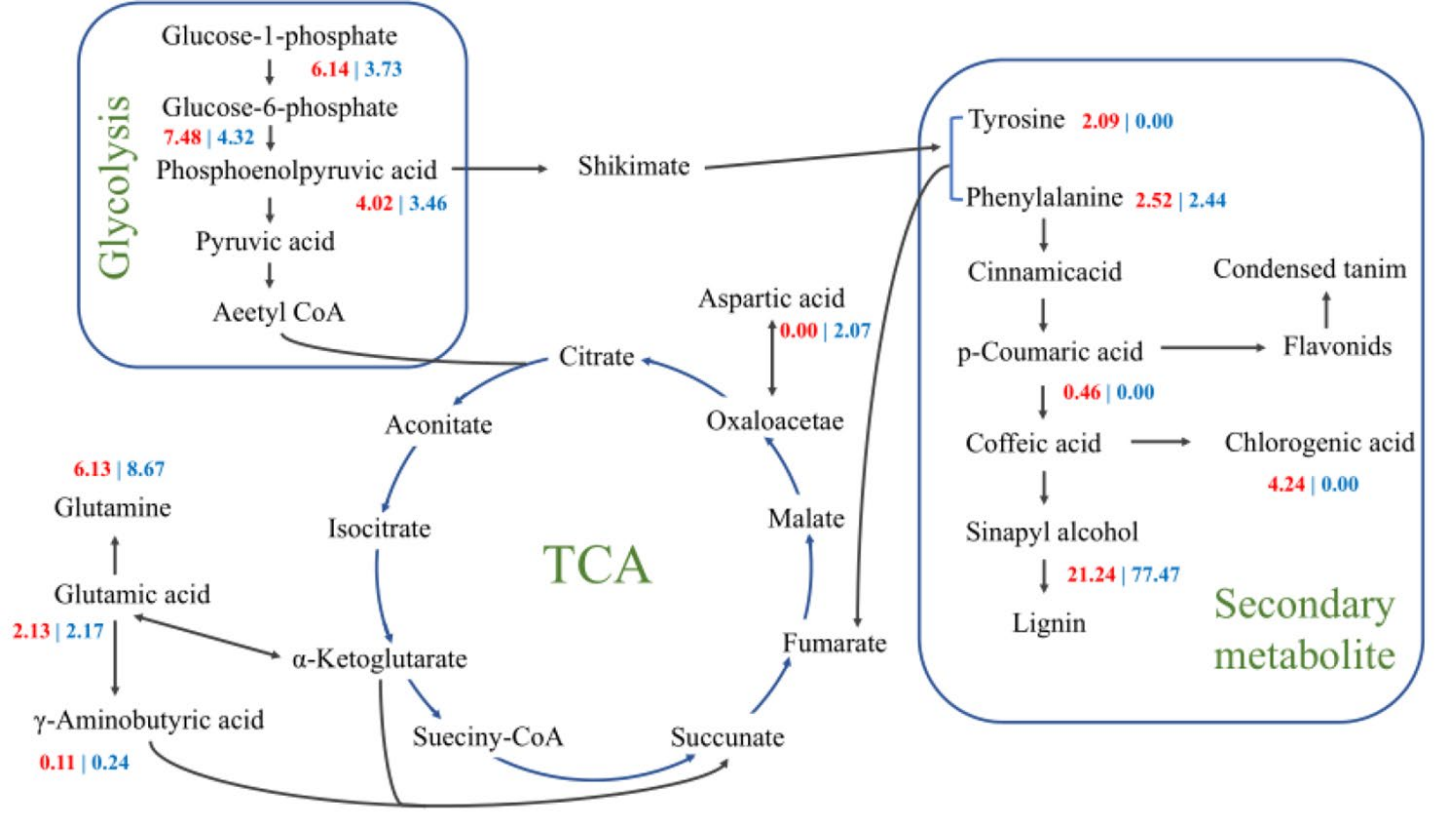
Maps of metabolic pathways involved in differentially expressed metabolites. These pathway maps mainly include glycolytic tricarboxylic acid cycle, amino acid biosynthesis, and secondary metabolism. The red color represents the fold change between nongerminated control and cold stress in N28. The blue color represents the fold change between nongerminated control and cold stress in N67

Plants usually consume more sugar and other energy carriers to maintain basic life activities under LT stress [35, 36]. Compared with nongerminated control, glycolysis intermediate products showed up-regulation under LT stress in N28 and N67 (Fig. 10), including glucose-1-phosphate, glucose 6-phosphate and phosphoenolpyruvic acid, but the fold change of N28 was greater than that of N67. Obviously, the sugar metabolism of N28 was more active than that of N67 under LT stress. Furthermore, there was no significant difference in the accumulation of phenylalanine and glutamic acid in N28 and N67. However, tyrosine and aspartic acid were differently accumulated in N28 and N67 (Fig. 10). Meanwhile, intermediate products of the shikimate pathway were differently accumulated in N28 and N67. The fold change of chlorogenic acid of N28 was greater than that of N67, but the fold change of sinapyl alcohol of N28 was smaller than that of N67. To sum up, these results indicated that N28 and N67 might employ different metabolic pathways to respond to LT stress.

### Comprehensive analysis of metabolome and transcriptome

**Fig. 11:**
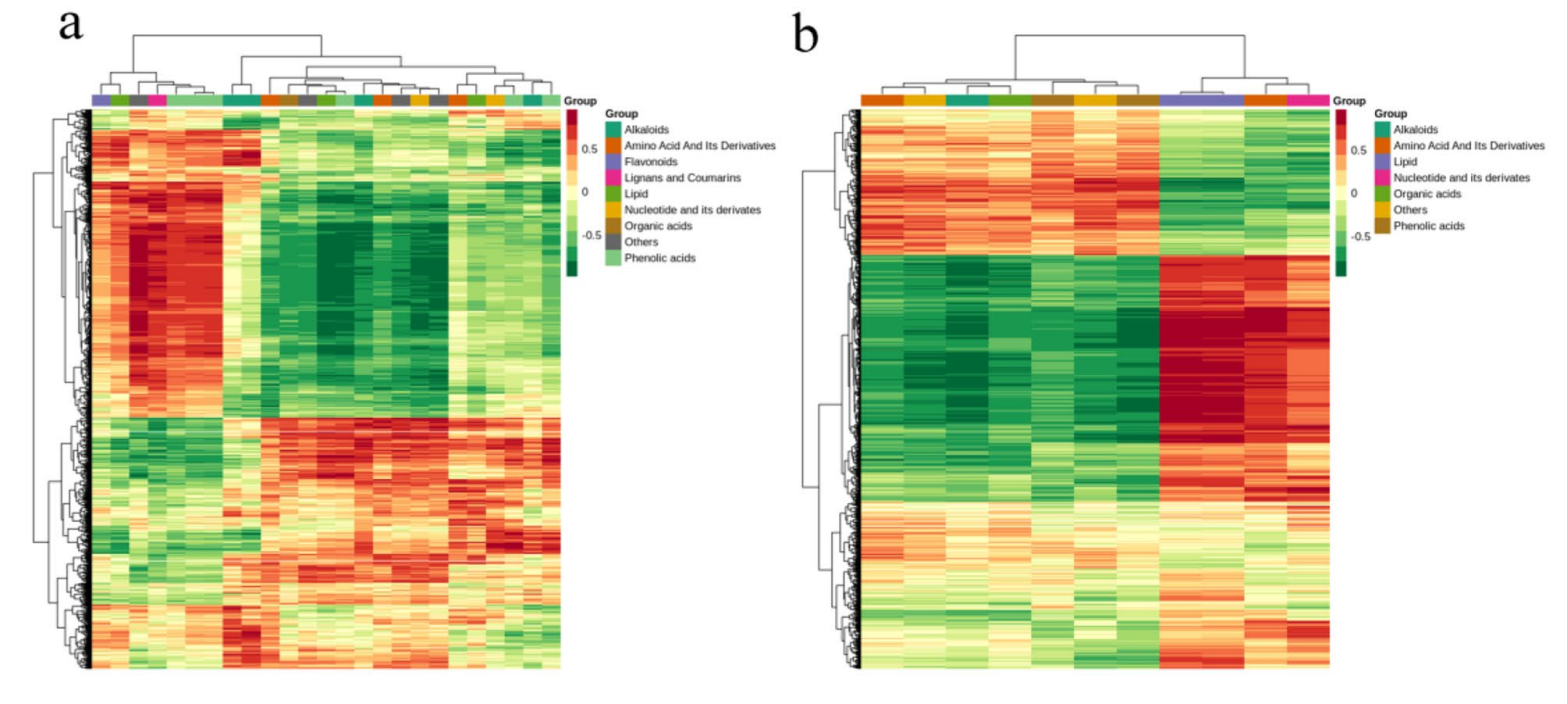
Heating map of correlation coefficient in N28 and N67. **a** Correlation analysis of DEGs and differential metabolites between nongerminated control and cold-stressed N28 samples. **b** Correlation analysis of DEGs and differential metabolites between nongerminated control and cold-stressed N67 samples. The abscissa represents the types of differential metabolites, and the ordinate represents DEGs

The combined analysis of transcription and metabolomics can help us to systematically and comprehensively study the function and regulation mechanism of biomolecules, and finally realize a comprehensive understanding of the trend and direction of biological changes [37]. In order to understand the correlation between differential metabolites and differential genes, we selected the results with Pearson correlation coefficient greater than 0.8 to conduct correlation analysis on differential genes and differential metabolites, and made a clustered heatmap (Fig. 11). We identified 2826 differentially expressed genes related to 9 types of metabolites between control and cold-stressed N28 samples. For N67, there were 1778 differentially expressed genes associated with 7 types of metabolites. Among them, flavonoids, lignans and coumarins were only found in N28, and these metabolites might give N28 higher cold resistance under LT germination.

## Discussion

### Low temperature affects physiological indexes of seed germination

Low temperature stress can affect a series of morphological and physiological changes of plants, and delay seed germination of maize, which has a great impact on its growth, yield and quality [38]. LT stress can increase the accumulation of reactive oxygen species (ROS), and the ROS scavenging enzymes in plants are mainly antioxidant enzymes, such as SOD, POD and CAT, etc [39]. In addition, the contents of proline (PRO) and soluble sugar (SS) in plant can be increased to improve the resistance to LT stress [40]. In this research, the content of SOD, POD, CAT, PRO and SS in N28 was significantly higher than that of N67. Electrical conductivity (EC) and the content of MDA can reflect the index of membrane permeability, and can be used to measure the degree of cold resistance of plants. The higher the EC and MDA, the more serious the plant is damaged by low temperature [17, 41]. In our research, the EC and the content of MDA in N67 is higer than that of N28. Therefore, the different resistance of N28 and N67 in low temperature may be due to the different activities of antioxidant enzymes and the different contents of osmoprotectants.

### Plant signal transduction pathway in response to LT stress

Phytohormones is important for plant to growth, development and metabolism. Previous studies have indicated that phytohormones play a critical role in helping plants adapt to adverse environmental conditions, and involved in the response of plants to LT stress [42–44]. Studies have shown that low temperature stress can affect plant growth and development. LT treatment at 4 °C can increased endogenous indoleacetic acid (IAA) content in rice, and cold stress strongly induced transcription levels of *OsYUCCA2*, *OsYUCCA3*, *OsYUCCA6* and *OsYUCCA7* [45]. In our study, plant signal transduction pathways were significantly enriched in both N28 and N67, and many genes related to auxin, gibberellin, ethylene, salicylic acid and abscisic acid were regulated under cold stress. Studies have shown that LT stress can induce an increase in ethylene content in plants [46, 47]. Ethylene as a signaling molecule, can promote the synthesis of antifreeze proteins and improve the cold resistance of winter rye [48]. The same result was found in our study. Compared with N67, the expression level of ETR, a negative regulator of ethylene synthesis, was significantly down-regulated in N28, which may be related to improved ethylene synthesis in seeds.

### MAPK signal pathway in response to LT stress

MAPK cascades reaction pathway is an important signal transduction pathway in plants. In this pathway, signals are transmitted through stepwise phosphorylation of MAPKKK, MAPKK and MAPK, and then downstream substrates are phosphorylated by MAPK to regulate the expression of corresponding genes and promote plant response to stress [49]. Previous studies have shown that AtMEKK1 gene can activate the expression of AtMPK4 and AtMPK6 by phosphorylation of downstream AtMEK1 and AtMKK2, so as to improve the cold resistance of Arabidopsis thaliana under low temperature stress [50]. In this study, MAPK signal transduction pathway - plant pathway is significantly enriched in both N28 and N67, and the expression levels of MEKK1 and MKK2 in MAPK pathway are significantly up-regulated under LT stress. It is concluded that MAPK signaling pathway plays a vital role in waxy corn that can resist LT stress. The three components of MAPK cascades pathway, with three components, can directly or indirectly participate in ABA signal transduction, and ABA signal can also regulate the expression level of related components genes in MAPK cascades pathway [51, 52]. MAK1 and MPK2 are activated by ABA in a SnRK2s dependent manner, establishing a direct link between ABA and MAPK [53]. In our study, it was found that under LT stress, the expression levels of most genes of PYR/PYLs and SnRK2s in ABA signaling pathway were significantly up-regulated, while the expression levels of all gene pairs of PP2Cs were significantly down-regulated. Therefore, waxy corn may use the interaction of MAPK cascade pathway and ABA signal transduction to improve cold resistance that can response to LT stress.

### Flavonoid metabolites in response to LT stress

Flavonoid metabolites as an important secondary metabolites, plants will accumulate a large amount of flavonoids when they under stress [54]. Flavonoids not only have ROS scavenging ability, but also can activate defense-related signaling pathways and regulatory mechanisms as signaling molecules [55]. Studies have showed that the content of flavonoids in Arabidopsis leaves was related to the strength of cold resistance [56]. Previous analysis was conducted by analysis of the transcriptome and metabolome of tobacco leaves after cold stress, and found that cold stress significantly affected phenylpropionic acid biosynthesis, resulting in a large amount of lignin and flavonoids accumulation, indicating that flavonoids play an important regulatory role in LT stress [57]. In this study, compared with N67, N28 significantly upregulated flavonoids and phenolic acid metabolites. Meanwhile, combined metabolome and transcriptome analysis showed that flavonoids, lignin and coumarin metabolites were only found in N28. Therefore, N28 can up-regulate the expression of flavonoid metabolites, which can improve N28 LT tolerance.

## Conclusions

In a word, we measured the phenotype and physiological indexes of two inbred lines, and found that the cold resistance of N28 was significantly higher than that of N67. To understand the cold resistance response mechanism, we compared the transcriptome and metabolome changes of N28 and N67 under LT stress.

The results showed that compared with N67, the expression level of some genes involved in plant hormones and MAPK signaling pathways was significantly up-regulated in N28, and flavonoid metabolites were also significantly enriched in N28 under LT germination. These genes and metabolites may help improve the cold resistance of N28 and may become potential target genes for breeding of cold-resistant waxy corn, but further research is needed.

## Methods

### Plant materials, temperature treatments and sampling

Seeds of two waxy corn inbred lines N28 and N67 were provided by Guangzhou Key Laboratory for Research and Development of Crop Germplasm Resources. N28, a low-temperature resistant waxy corn, which is the paternal parent (♂) of waxy corn variety Zhongnuo No.1, and N67, a LT-sensitive waxy corn, which is the paternal parent (♂) of waxy corn variety Zhongnuo No.8. The seed surface of both waxy corn varieties were sterilized, washed with sterile water, and germinated in sterile petri dishes that contained wet filter paper under normal temperature (NT) conditions (25 °C) and LT conditions (15 °C) respectively, with three replicates of 90 seeds per treatment. Petri dishes were placed in an artificial climate box, with a photoperiod of 16 h light and 8 h dark.

For physiological index measurement, 300 seeds of N28 are divided into 3 replicates and then subjected to germination test under NT, and another 300 N28 seeds were germinated in the same way under LT. Germination test of N67 was conducted with the same method. The number of seeds germinated was recorded daily during the germination process till the number of germination no longer changed (the 7th day after germination). At the 7th day after germination, 10 seedlings were selected randomly from each replicate to measure root length, bud length and seedling fresh weight and dry weight after drying, then calculated the mean of each index.

For physiological index measurement, 300 seeds of N28 are divided into 3 replicates and then subjected to germination test under LT, and 300 N67 seeds were germinated in the same way. Each sample including 10 germinating seed was collected after 0, 24, 48, 72 and 96 h of germination under LT.

For metabolomics and transcriptomics analysis, 90 seeds of N28 are divided into 3 replicates and then subjected to germination test under NT, and another 90 N28 seeds were germinated in the same way under LT. Germination test of N67 was conducted with the same method. After 3 days of germination, when the seed radicle of N67 under LT broke through the seed coat, Each samples including 5 germinating seed were harvested for N28 and N67 under NT and LT conditions, respectively. Nongerminated dry seeds were used as control. All seed samples were immediately frozen in liquid nitrogen and stored at -80 °C.

### Phenotype analysis of germinated waxy corn seeds under LT

To further study the dynamic response of N28 and N67 during germination under LT stress, we analyzed some phenotypic indexes of the two inbred lines. The number of seeds germinated was recorded daily during the germination process till the number of germination no longer changed, and the relevant germination index was calculated. N67 and N28 were detected for germination rate (GR), germination energy (GE), germination index (GI), vitality index (VI), root length (RL), bud length (BL), fresh weight (FW) and dry weight (DW) under NT and LT germination conditions. SPSS was used to analyze the significance of differences and the variance between samples.

### Measurement of soluble sugar, proline, malonaldehyde and protein

The ground samples were dissolved in PBS or ultrapure water, and the supernatant was collected by centrifugation. The content of soluble sugar, proline (PRO), malonaldehyde (MDA) and protein was detected using ultraviolet spectrophotometry (MAPADA, UV-1200), respectively. For the soluble sugar determination, under the action of concentrated sulfuric acid, sugar can be dehydrated to produce furfural or hydroxymethyl furfural, and the product can react with anthrone to produce blue-green furfural derivatives. The absorbance was detected at 620 nm. For the PRO determination, PRO reacted with acidic ninhydrin to produce a stable red product, forming a characteristic absorption peak at 520 nm. For the MDA determination, MDA can condense with thiobarbituric acid (TBA) to form a red product with a maximum absorption peak at 532 nm. For the protein determination, coomassie brilliant blue G-250 combined with protein to form a characteristic absorption peak at 595 nm.

### Electronic conductivity and enzyme activity assays

The ground samples were dissolved in PBS and the supernatant was collected by centrifugation. The content of superoxide dismutase (SOD), peroxidase (POD) and catalase (CAT) was detected using ultraviolet spectrophotometry (MAPADA, UV-1200), respectively. SOD was quantified using SOD assay kit (Nanjing Jiancheng, China), POD was quantified using PODassay kit (Nanjing Jiancheng, China), CAT was quantified using CAT assay kit (Nanjing Jiancheng, China). For the electronic conductivity (EC) determination, 50 seeds of uniform size were rinsed 3 times with distilled water, and the surface water was absorbed by filter paper. Each sample was put into an Erlenmeyer flask with 250mL of distilled water to determine the conductivity.

### Library preparation and RNA-sequencing

Total RNA was isolated using TRIzol reagent (Invitrogen) according to the manufacturer’s instructions. RNA purity, concentration and integrity were checked, using the NanoPhotometer®spectrophotometer (IMPLEN, Westlake Village, CA, USA), Qubit ® RNA Assay Kit in Qubit®2.0 Flurometer (Life Technologies, Carlsbad, CA, USA)and RNA Nano 6000 Assay Kit of the Agilent Bioanalyzer 2100 system (Agilent Technologies, SantaClara, CA, USA), respectively. The mRNA with polyA tail was enriched with oligo magnetic beads, then purified. The cleaved RNA fragments were reverse-transcribed to double-strand cDNA using N6 random primer. The cDNA fragments were purified, blunted with phosphate at 5’end and stickiness “A” at 3’end, and adaptor-ligated. These products were subsequently purified and amplified by PCR to create cDNA libraries. Finally, the cDNA libraries were sequenced on the Illumina HiSeq sequencing platform.

### Analysis of transcription factors and differentially expressed genes (DEGs)

Transcription factors (TF) were predicted using iTAK software and identified by hmmscan comparison [58]. DESeq2 was used to perform differential expression analysis between sample groups. The false discovery rate (FDR) was deliberately controlled by the adjusted *P*-value, which were adjusted by Benjamini and Hochberg’s approach. *P*-value < 0.05 was set as a threshold for significantly enriched categories.

### GO term and KEGG pathway enrichment

The CDS sequence of the gene was annotated to Kyoto Encyclopedia of Genes and Genomes (KEGG) and Gene Ontology (GO) using the BLASTX analysis with a cut-off E-value of 10^− 5^. GO analysis was conducted using the gene set enrichment analysis base (GSEABase) package from BioConductor (http://www.bioconductor.org/) based on biological process categories (Fisher’s exact test, FDR < 0.001). Pathway enrichment analysis was conducted to illustrate significant pathways of DEGs according to KEGG (http://www.genome.jp/kegg) databases [59, 60].

### Quantitative real-time PCR

Total RNA was reverse-transcribed usingPrimeScript™ RT reagent Kit with gDNA Eraser (Takara Bio Inc., Otsu, Japan). The qRT-PCR assay was completed with 2×SYBR Premix Ex Taq™ II (Takara Bio Inc., Otsu, Japan) and the CFX96 Real-Time PCR Detection System (Bio-Rad Laboratories, Inc., USA). An actin gene was used as a reference control. The two-step PCR program was as follows: 94 °C for 30 s; 40 cycles of 94 °C for 5 s and 60 °C for 30s. Finally, a melting curve analysis was completed under the following conditions: 95 °C for 10 s, 65 °C for 5 s, 0.5 °C/s up to 95 °C. The relative fold-changes of gene expression were calculated using the comparative 2^−ΔΔCT^ method [61]. All samples were analyzed with three technical and biological replicates. The primers used for qRT-PCR analysis are listed in Table 4.

**Table 4:** Primers for real-time quantitative PCR of selected DEGs

Gene	Forward primer (5′ to 3′)	Reverse primer (5′ to 3′)
Zm00001d005892	CAGTCCAATGATCGGAGGAT	GCTGTACCCGGACACCAC
Zm00001d043179	CCGAACACCTATGCCAGTTT	GCTATGAACGTCCCTGAAGC
Zm00001d005890	ACCACGTTTTCGACTGGAAC	GCTTCTCCCAAACGCAGTAG
Zm00001d048709	TACAAGCCCATCATGTCTCG	GCTCACCAGGTAGACGAAGC
Zm00001d016031	GAGGGGCATCGATAAACTGA	CAGGTCGTTGAAGCTGTTGA
Zm00001d007372	CAGCGAGCTCCTTCATTACC	CGGCCTCCACGAAGTAGTAG
Zm00001d002288	GATCTGCCGAGTTCCACAAT	GGAACCACTGGTGACATTCC
Zm00001d001837	ATCTCGCGGAACTTTGTGAT	GATTTGCAGCCTGACCATTT
Zm00001d005890	ACCACGTTTTCGACTGGAAC	GTGGAAGTCTGAGGGGATCA
Zm00001d007827	CATGGTGGGCTTCGTGTC	CGGTGAGGTACTCAGTGAAGG
Zm00001d051362	CTGACGGGCGTCTTCTACTG	AGGTGATGACGACCTCGAAC
Zm00001d028714	GCTACAATGCGAAGGTAGGC	AGGCGTGGTACTGGTGGTAG
Zm00001d052335	CTGTCCACGGGCTTCTACTC	TGTTAGGGTTGGCCATCTTC
Zm00001d001850	ACCTGATCGTTGGAAAGGTG	GTCGCTTCCCCTGTACACAT
Zm00001d024317	CGTTGCCAGCTTCAGAAAGT	GTGGAAGGAGAAGGACACCA
Zm00001d017996	CAGGACGTGTGCAAGGACTA	AGTCGGTGAAGAAGGCAGTG
Zm00001d025676	TCCGAAGGGTAAGGTGTTTG	GTTACAACGTCCAGCCCTGT
Zm00001d032821	CGGTAATGGCGGGTCTACTC	CTAGCCTCACTAGAGCCGGA
Zm00001d027291	CTCCTCAACTACTGCGGCAA	AAAACATCCGGAGGAGGCAC
Zm00001d016256	TTGCAGTTGTTGGGTCCCTT	GTCAGAGGTCACGGACACAG

### Sample preparation and metabolite extraction

The freeze-dried samples were then crushed with a mixer mill (MM 400, Retsch, Haan, Germany) with a zirconia bead for 1.5 min at 30 Hz. The powder (100 mg) was weighed and extracted overnight at 4 °C with 1.0 mL of 70% aqueous methanol. The supernatant was collected by centrifugation at 10,000 g for 10 min. Finally, the extracts were absorbed (CNWBOND Carbon-GCB SPE Cartridge, ANPEL) and filtered by 0.22 μm filters (SCAA-104, ANPEL) for LC-MS analysis.

### Metabolic profiling analysis

The extracts were analyzed using a Ultra Performance Liquid Chromatography system(Shim-pack UFLC SHIMADZU CBM30A, https://www.shimadzu.com.cn/)couple*d* with a tandem mass spectrometer(Applied Biosystems 4500 QTRAP, http://www.appliedbiosystems.com.cn/*).* The experiment conditions were as follows: the UPLC system was equipped with ACQUITY UPLC HSS T3 C18 column (Waters, 1.8 μm×2.1 mm×100 mm); solvent system, water (0.04% acetic acid) and acetonitrile (0.04% acetic acid); gradient program (95:5 v/v at 0 min, 5:95 v/v at 11.0 min, 5:95 v/v at 12.0 min, 95:5 v/v at 12.1 min, 95:5 v/v at 15.0 min with the flow rate of 0.40 mL/min); column temperature (40 °C); injection volume (4 µL). The effluent was connected to an ESI-triple quadrupole linear ion trap mass spectrometer (ESI-QTRAP-MS).

Linear ion trap (LIT) and triple quadrupole (QQQ) scans were acquired on QTRAP equipped with an ESI Turbo Ion-Spray interface in the positive ion mode and controlled by Analyst 1.6.3 software (AB Sciex, Framingham, MA, USA). The ESI source operation followed Chen’s parameters [62]: ion source, turbo spray, source temperature 550 °C, ion spray voltage (IS)5500 V, ion source gas I (GSI), gas II(GSII), and curtain gas (CUR) were set at 55, 60, and 25.0 psi, respectively, the collision gas (CAD) was high. Instrument tuning and mass calibration were performed with 10 and 100 µmol/L polypropylene glycol solutions in QQQ and LIT modes, respectively. The QQQ scans were acquired as multiple reaction monitoring (MRM) experiments with the collision gas (nitrogen) set to 5psi. The declustering potential (DP) and collision energy (CE) for individual MRM transitions were determined with further DP and CE optimization [63]. A specific set of MRM transitions were monitored for each period according to the metabolites eluted within this period.

### Qualitative and quantitative analysis of metabolites

On the basis of a self-established database (MWDB) and a public database concerning metabolite information, the MRM mode of the QQQ mass spectrometer was used for metabolite qualitative and quantitative analysis. The qualitative analysis of metabolites was conducted using the stepwise multiple ion monitoring-enhanced product ions (MIM-EPI) strategy. For the quantitative analysis, the area of all the acquired mass spectrum peaks was calculated. The mass spectral peaks detected from each metabolite were corrected on the basis of information on metabolite Rt and peak type in order to ensure the precision of the qualitative and quantitative analyses.

## Electronic supplementary material

Below is the link to the electronic supplementary material.

Supplementary Material 1

Supplementary Material 2

## Data Availability

All datasets generated or analyzed during this study are available from the corresponding author upon reasonable request. The raw transcriptome data has been submitted to NCBI SRA under the accession number: PRJNA911416 (https://www.ncbi.nlm.nih.gov/bioproject/PRJNA911416).
