# Supplementary material for: Integrated metabolomics and transcriptomics analysis during seed germination of waxy corn under low temperature stress
Source: BMC Plant Biol. 2023 Apr 10;23:190. doi: 10.1186/s12870-023-04195-x (PMC10084618; doi:10.1186/s12870-023-04195-x)
Supplement: Supplementary file 1 — Supplementary Material 1 [file 12870_2023_4195_MOESM1_ESM.docx]

**Additional file 1: Table S1**. N28 and N67 seed germination phenotype analysis

| Inbred lines |  | N28 | N67 |
| --- | --- | --- | --- |
| GR (%) | NT | 100.00±0.00 a | 95.33±5.13 a |
|  | LT | 90.02±10.03 a | 53.33±15.28 b |
| GE (%) | NT | 71.11±6.94 a | 67.78±6.94 a |
|  | LT | 70.02±10.04 a | 23.33±15.28 b |
| GI | NT | 19.48±2.54 a | 19.29±1.24 b |
|  | LT | 3.69±0.56 a | 1.48±0.82 b |
| VI | NT | 91.69±26.71 a | 83.23±17.77 a |
|  | LT | 3.11±1.03 a | 0.25±0.01 b |
| RL (cm) | NT | 7.05±3.72 a | 7.78±4.62 a |
|  | LT | 2.77±1.08 a | 1.19±0.39 b |
| BL (cm) | NT | 4.66±1.28 a | 4.29±1.34 a |
|  | LT | 0.83±0.25 a | 0.25±0,09 b |
| FW (g) | NT | 0.55±0.03 a | 0.63±0.14 a |
|  | LT | 0.40±0.03 a | 0.12±0.04 b |
| DW (10-3g) | NT | 320.05±36.06 a | 433.33±100.66 a |
|  | LT | 57.03±7.28 a | 7.97±1.55 b |

Note: The values in the table are expressed as mean ± standard deviation (n=3), there is no significant difference between the same letters at the same temperature (P>0.05).
