# Supplementary material for: Integrated metabolomics and transcriptomics analysis during seed germination of waxy corn under low temperature stress
Source: BMC Plant Biol. 2023 Apr 10;23:190. doi: 10.1186/s12870-023-04195-x (PMC10084618; doi:10.1186/s12870-023-04195-x)
Supplement: Supplementary file 2 — Supplementary Material 2 [file 12870_2023_4195_MOESM2_ESM.docx]

**Additional file 2: Table S2** Quality control analysis of transcriptome data

| **Sample** | **Raw Reads** | **Clean Reads** | **Error Rate (%)** | **Q20 (%)** | **Q30 (%)** | **GC Content (%)** |
| --- | --- | --- | --- | --- | --- | --- |
| MC28-1 | 52067554 | 51349714 | 0.02 | 97.16 | 92.46 | 57.11 |
| MC28-2 | 45761798 | 44622064 | 0.02 | 96.93 | 91.96 | 56.83 |
| MC28-3 | 47688786 | 46355268 | 0.02 | 96.99 | 92.09 | 57.31 |
| MC67-1 | 76320654 | 75094276 | 0.02 | 97.92 | 94.10 | 59.49 |
| MC67-2 | 71092782 | 69821772 | 0.02 | 97.86 | 94.01 | 58.83 |
| MC67-3 | 74114382 | 73097604 | 0.02 | 98.04 | 94.38 | 58.98 |
| MN28-1 | 52340970 | 51526680 | 0.02 | 97.77 | 93.61 | 55.12 |
| MN28-2 | 53385010 | 52515246 | 0.02 | 98.03 | 94.19 | 55.20 |
| MN28-3 | 55778728 | 54827466 | 0.02 | 97.72 | 93.48 | 55.64 |
| MN67-1 | 48991694 | 48029774 | 0.02 | 96.72 | 91.50 | 55.63 |
| MN67-2 | 49552222 | 48520670 | 0.02 | 97.22 | 92.48 | 55.38 |
| MN67-3 | 61092446 | 59962440 | 0.02 | 97.77 | 93.54 | 55.66 |
| ML28-1 | 57673336 | 56792122 | 0.02 | 97.91 | 93.89 | 54.76 |
| ML28-2 | 43880478 | 42902030 | 0.02 | 96.93 | 91.86 | 54.58 |
| ML28-3 | 53655558 | 52249714 | 0.02 | 97.72 | 93.45 | 54.73 |
| ML67-1 | 63886680 | 62862078 | 0.02 | 97.84 | 93.79 | 55.13 |
| ML67-2 | 51918808 | 50969042 | 0.02 | 97.81 | 93.69 | 55.57 |
| ML67-3 | 46642366 | 45892004 | 0.02 | 97.78 | 93.61 | 54.98 |

MC28 and MC67 refer to the nongerminated control samples of N28 and N67; MN28 and MN67 refer to N28 and N67 samples germinated at NT; ML28 and ML67 refer to N28 and N67 samples germinated at LT, respectively; 1,2 and 3 refer to the three replicates
